# A Novel Computational Approach for the Discovery of Drug Delivery System Candidates for COVID-19

**DOI:** 10.3390/ijms22062815

**Published:** 2021-03-10

**Authors:** Taeheum Cho, Hyo-Sang Han, Junhyuk Jeong, Eun-Mi Park, Kyu-Sik Shim

**Affiliations:** 1 MODNBIO Inc., Digital Road 34, Kolon Science Valley I, Guro-gu, Seoul 08378, Korea; taeheumcho89@modnbio.com (T.C.); jjh93@modnbio.com (J.J.); pem2019@modnbio.com (E.-M.P.); 2 Department of Health Administration, Joongbu University, Chungnam 31713, Korea; hanhs@joongbu.ac.kr

**Keywords:** COVID-19, in silico, machine learning, clustering, unsupervised learning, drug delivery system, nafamostat, computer-aided drug discovery, CADD, docking, micelle nanoparticles

## Abstract

In order to treat Coronavirus Disease 2019 (COVID-19), we predicted and implemented a drug delivery system (DDS) that can provide stable drug delivery through a computational approach including a clustering algorithm and the Schrödinger software. Six carrier candidates were derived by the proposed method that could find molecules meeting the predefined conditions using the molecular structure and its functional group positional information. Then, just one compound named glycyrrhizin was selected as a candidate for drug delivery through the Schrödinger software. Using glycyrrhizin, nafamostat mesilate (NM), which is known for its efficacy, was converted into micelle nanoparticles (NPs) to improve drug stability and to effectively treat COVID-19. The spherical particle morphology was confirmed by transmission electron microscopy (TEM), and the particle size and stability of 300–400 nm were evaluated by measuring DLSand the zeta potential. The loading of NM was confirmed to be more than 90% efficient using the UV spectrum.

## 1. Introduction

Declared as a global pandemic by the World Health Organization (WHO) in 2020, the 2019 novel coronavirus has created serious problems in healthcare systems and daily life worldwide. Nafamostat mesilate (NM) is a serine proteinase inhibitor that has been used in Japan to treat disseminated intravascular coagulation (DIC) and pancreatitis for over 30 years [1,2] and has been used in the treatment of Coronavirus Disease 2019 (COVID-19). NM blocks the SARS-CoV-2 S protein-initiated fusion by inhibiting protease in 293FTcells (derived from human fetal kidney) ectopically expressing angiotensin converting enzyme 2 and transmembrane serine protease 2 (TMPRSS2). When conducting similar experiments with Calu-3 cells, which are considered a suitable model for human SARS-CoV-infected cells, NM significantly inhibited membrane fusion at low concentrations in the range of 1–10 nM. This is almost the same as the concentration range of membrane fusion inhibition by the MERS-CoV S protein [3,4]. Despite this significant efficacy, NMs have very poor stability in the body, so the molecular structure needs to be regenerated/stabilized to promote efficient drug delivery and treatment. Therefore, we developed a drug delivery system (DDS) using micelle NPs that can compensate for these limitations and maximize the effectiveness of NM. When NM is stabilized with micelle NPs, it has been shown that it can prevent degeneration, inhibit infection by COVID-19, and treat infected cells after intracorporeal administration. As a low-molecular weight compound harmless to the living body, if NM is loaded on micelle NPs, it becomes a nanoparticle-sized formulation, thereby inhibiting the TMPRSS2 receptor by maintaining its own stability, and hence is expected to treat or prevent COVID-19. While the existing NM administration method is a difficult infusion method, it appears to be able to be administered orally. To manufacture ideal micelle NPs that can act as DDS, they must have an amphiphilic structure and have a structure whose hydrophobic length is slightly shorter than that of hydrophilic [5]. Spherical micelles were prepared using hydrophilic and hydrophobic structures of different lengths. In addition, the possibility of micelle formation was confirmed in a triblock (long hydrophilic—hydrophobic—short hydrophilic) structure to load a hydrophilic drug [6]. For the purpose of searching for the candidates satisfying this condition, we developed a novel computational method. This was tested with a drug database provided by DrugBank 5.0 (https://go.drugbank.com/, accessed on 8 March 2021) that has been employed in many drug screening studies for drug repositioning as it consists of approved small molecule drugs and biologics, nutraceuticals, and experimental drugs. Drug repositioning (also called repurposing) means reusing an approved drug for another purpose, so its safety and production methods have already been confirmed [7].

Repositioning/repurposing a drug would reduce the time and cost associated with development, as several stages spent in the development for its original indication could be avoided [8,9]. Drug delivery systems can be a vital tool for repositioning and have been studied in recent years [10,11]. However, experimental confirmation of DDS is not an easy task because in vitro experiments are arduous and time-consuming. To overcome these limitations, several computational approaches, such as deep learning (DL) and machine learning (ML) methods, have been suggested [12]. For example, YuanYuan et al. [13] developed a data-driven predictive system based on ML techniques to determine the effectiveness of drug dosing in drug delivery methods. Mikolaj et al. [14] presented quantitative structure–property relationship (QSPR) models that predict the stability of the complexes formed by a popular, poorly soluble antibiotic, cefuroxime axetil, and different cyclodextrins. The proposed method is relevant in structure-based drug design (SBDD), which identifies leads suitable for clinical testing through molecular structure, although not common. As our candidate material uses substances registered in the drug bank, micelle NPs enclosing the drug not only act as a carrier of the drug using the DDS, but are themselves a dual mode of drug action that can exert its efficacy.

## 2. Results

### 2.1. Results of Carrier Suitability Scoring Method

**Figure 1:**
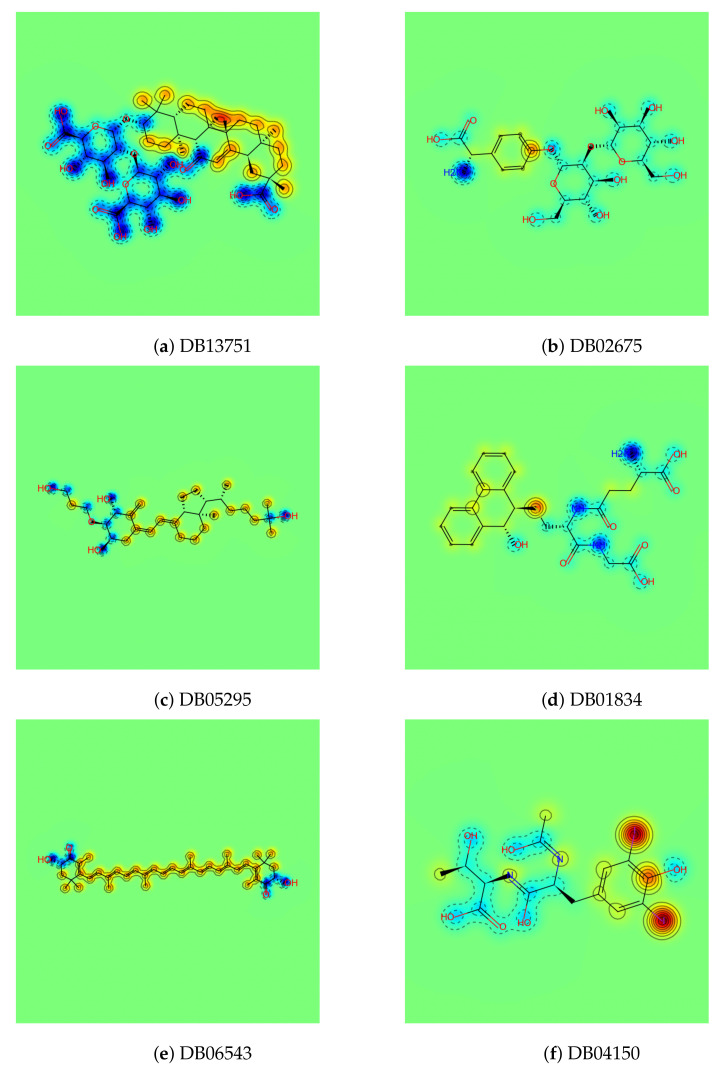
Six final carrier candidates found by the CSS method and qualitative examination. The color on the similarity maps represents the atom-wise partition coefficient logP: the blue area indicates the hydrophilic atoms having low logP, and the red indicates the hydrophobic atoms having a high logP.

In Table 1, we list the molecules measured by the CS score. It is clear that the CSS method is good at finding molecules meeting predefined conditions. There are five sub-scores that make up the CS score, among which the first four (1, 2, 3, and 4) are structural scores and one (logP) is a chemical score. The description of each sub-score is given in Section 3.4. As mentioned before, six molecules were selected as carrier candidates through the CSS method, and qualitative examination using domain knowledge was performed. For instance, DB13751 obtained the third highest CS score, but was ranked the highest after passing the qualitative examination. This molecule also acquired the highest values of Scores 3 and 4. The first is the asymmetry between the size of the hydrophilic clusters; the larger the value of this score, the greater the difference in size between the two hydrophilic group clusters is. The second is whether the furthest atomic pair is included in the hydrophilic groups, and the value of two means that both atoms are included in the hydrophilic groups, while a value of zero means that neither are included. As shown in Figure 1a, the sizes of the two hydrophilic groups are asymmetrical, and the farthest atomic pairs also belong to hydrophilic groups. The greatest logP score was achieved by DB01834, the closer the logP is to zero, the greater the logP score. In Figure 1, the closer the color of the atoms in the molecule to blue or red, the stronger the atoms are considered to be hydrophilic or hydrophobic. As seen in Figure 1d, most of the areas in DB01834 are not blue or red, but light blue or yellow. In addition, the hydrophilic and hydrophobic areas within the molecule were similar in width. Hence, DB01834 had the highest logP score. DB06543 obtained the greatest value of Score 1, representing the distance between the hydrophilic group clusters. A high score of one indicates that two hydrophilic group clusters are well separated. It was observed that the hydrophilic group clusters are extremely far away.

The CSS method offers an intuitive way to inspect atom-wise partition coefficient maps of molecules, as shown in Figure 1. Of the 10,630 molecules, six molecules were nominated as carrier candidates after qualitative inspection with the naked eye of the molecules with the top 1.5% carrier suitability (CS) score. These maps were calculated using RDKit (http://www.rdkit.org/, accessed on 8 March 2021) [15]. We can see that the larger hydrophilic group cluster and the smaller one are separated well, and the triblock form of molecules is clearly represented. As is clearly visible in Figure 1, the proposed CSS method has excellent performance in finding the carrier candidates meeting our proposed conditions.

### 2.2. Results of Single Layer Modeling

**Figure 2:**
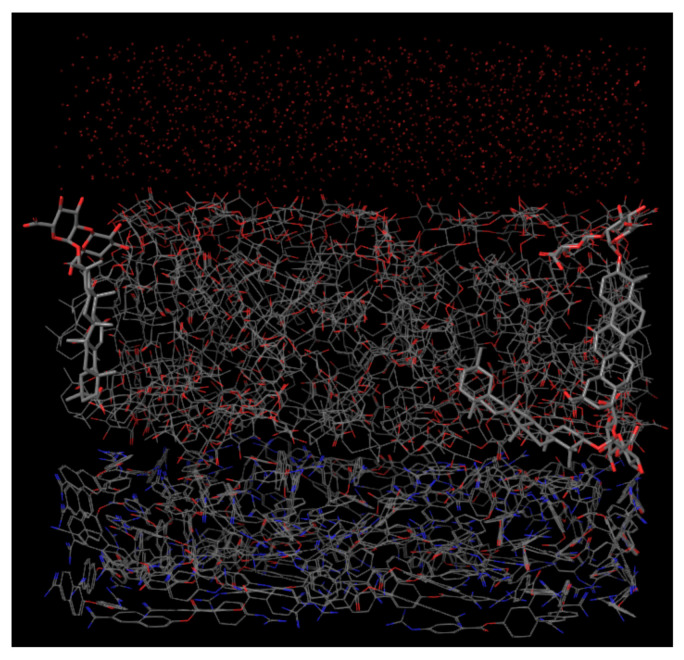
Result of single layer modeling of DB13751 between water and nafamostat performed by using the Schrödinger software. The result shows that a single layer of DB13751 was formed with some molecules of DB13751 arranged vertically with their hydrophilic heads directed to water (top) and nafamostat (bottom).

As can be seen in Figure 2, a single layer of DB13751 is formed between the water and nafamostat. It is identified that some DB13751 are aligned with their hydrophilic portions directed to water and nafamostat. The results of the modeling support the proposition that DB13751 can be a good candidate for drug delivery carriers. However, the simulation, being a virtual experiment, should not be regarded as verifying the conclusions of the paper.

### 2.3. Experimental Results (Evaluation Results)

#### 2.3.1. Characterization of NM-Loaded Micelle NPs

**Figure 3:**
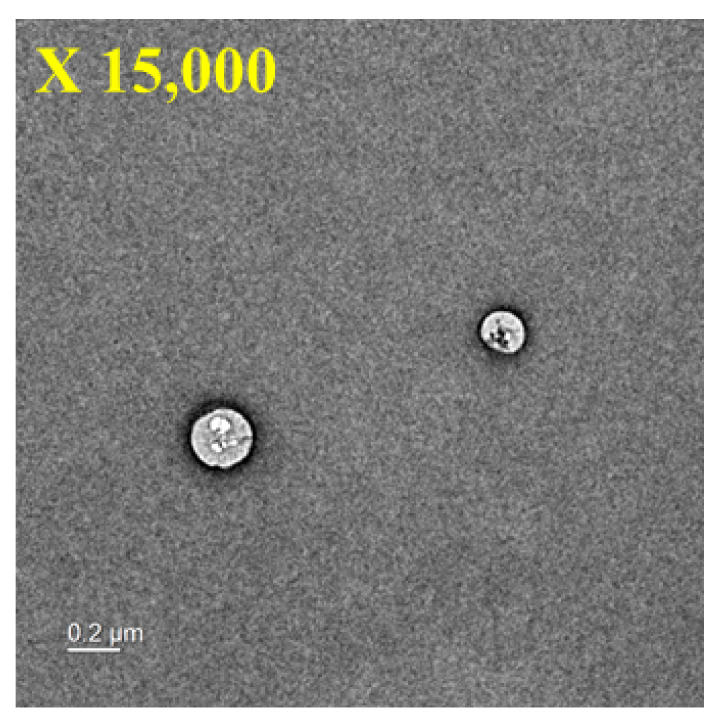
TEM images of NM-loaded micelle NPs.

**Figure 4:**
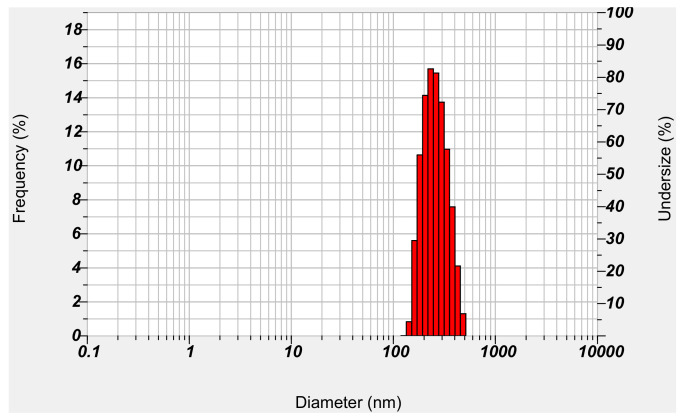
Particle size and distribution of NM-loaded micelle NPs.

**Figure 5:**
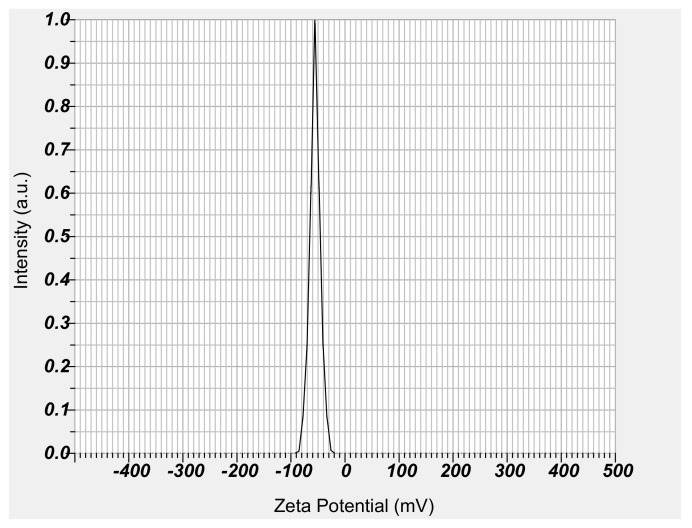
Zeta potential of NM-loaded micelle NPs.

Micelles formed with DB13751 were expected to have stable DDS effects while stably surrounding NM. After dissolving DB13751 in ethanol, oil was added to adjust the critical micelle concentration to form micelles. After that, nafamostat was loaded into the micelle through sonication, and the desired NM-loaded micelle NPs were obtained through lyophilization. The morphologies of NM-loaded micelle NPs when examined by TEM were spherical, as shown in Figure 3. The particle sizes and distributions of NM-loaded micelle NPs were determined by dynamic light scattering. The particle size of NM-loaded micelle NPs was approximately 394.1 ± 11.81 nm. Figure 4 shows that our NM-loaded micelle NPs showed a constant size. The confirmed zeta potential value was −55.5 ± 0.75 mV for NM-loaded micelle NPs, as shown in Figure 5. As a result, it can be seen that our NM-loaded micelle NPs stably formed micelles.

#### 2.3.2. Loading Amount of Nafamostat Mesilate

Micelles formed with DB13751 stably surrounded NM and improved the limitations of NM, and a stable DDS effect was expected. The loaded amounts and efficiencies of NM per 10 mg of NM-loaded micelle NPs were 759.9 ± 20.55 µg (92.24 ± 2.50%), respectively, for NM-loaded micelle NPs. It was confirmed that micelle NPs were manufactured with micelle-shaped NPs and produced with high efficiency as desired when NM was loaded. The amount of NM to be loaded will be controlled by research conducted later. In the future, studies performed with HPLC, NMR, MALDI-TOF MS, etc., will be added.

#### 2.3.3. Cell Cytotoxicity

**Figure 6:**
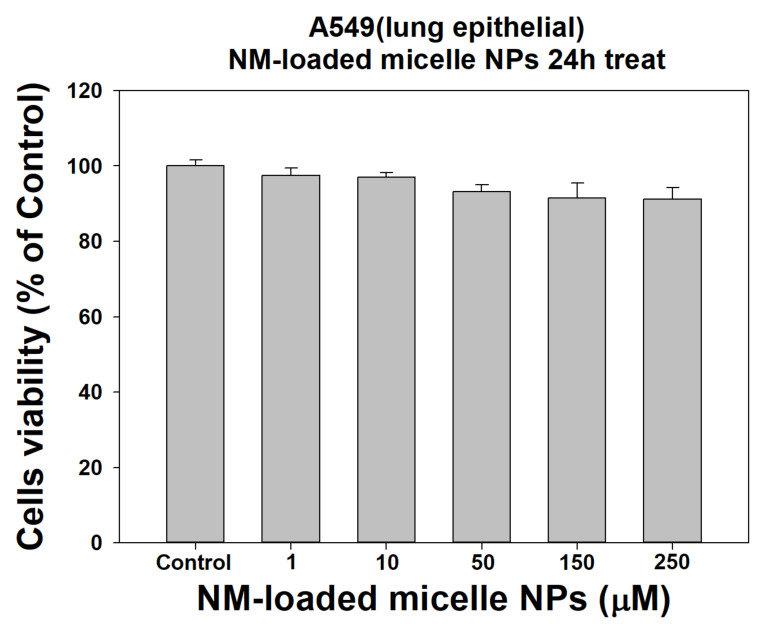
Cytotoxicity of control and NM-loaded micelle NPs (1, 10, 50, 150, and 250 µM) against A549 cells at one day (*n* = 8).

The concentration is based on nafamostat, and the concentration at which nafamostat is effective in cells is usually about 1 nM to 30 µM. Therefore, it was confirmed that there was no toxicity at the low concentration that could exert the effect, and the cell viability was more than 90%, even at the high concentration, so there was almost no toxicity. As a result, as shown in Figure 6, no cytotoxicity was observed at concentrations below 10 µM. It was found to be absent, and it was confirmed that there was almost no toxicity even at concentrations of 50 µM 250 µM. It was confirmed that the cytotoxicity of the nanoparticles was very low.

Based on the results, NM-loaded micelle NPs were constructed to analyze them. After fabrication, the nanoparticle size and stability were confirmed through DSLand the zeta potential, and the morphology of the particles was confirmed by TEM imaging. In addition, the NM loading efficiency was measured by ELISA, and it was confirmed that NM was well loaded in NM-loaded micelle NPs. NM-loaded micelle NPs were produced, and it was confirmed that the expected results were consistent with those of the CSS method. Cytotoxicity was performed to confirm that the prepared NM-loaded micelle NPs were non-toxic, and it was confirmed that there was no toxicity even at the desired concentration and a high concentration. Based on this, we plan to explore and develop drugs and carriers suitable for each disease using our technology as a platform. NM-loaded micelle NPs also target the virus causing COVID-19, and we will conduct in vitro and in vivo experiments for the characterization and efficacy of the substance. Glycyrrhizin is a substance known to inhibit the replication of RNA viruses [16]. When nafamostat is loaded onto micelle NPs and stabilized and delivered to cells, it is expected that the two constituents of the particle will show synergistic inhibitory efficacy against viral infection through a dual mode of action.

## 3. Materials and Methods

In silico analysis for deriving the carrier candidates is introduced in this section. This consists of two parts: the carrier suitability scoring (CSS) method and the Schrödinger simulation. For this study, DrugBank 5.0 (https://go.drugbank.com/, accessed on 8 March 2021) was used as a resource to obtain 10,630 samples of molecules for analysis. DrugBank is a web-accessible database consisting of comprehensive molecular information about drugs and their mechanisms [8].

**Figure 7:**
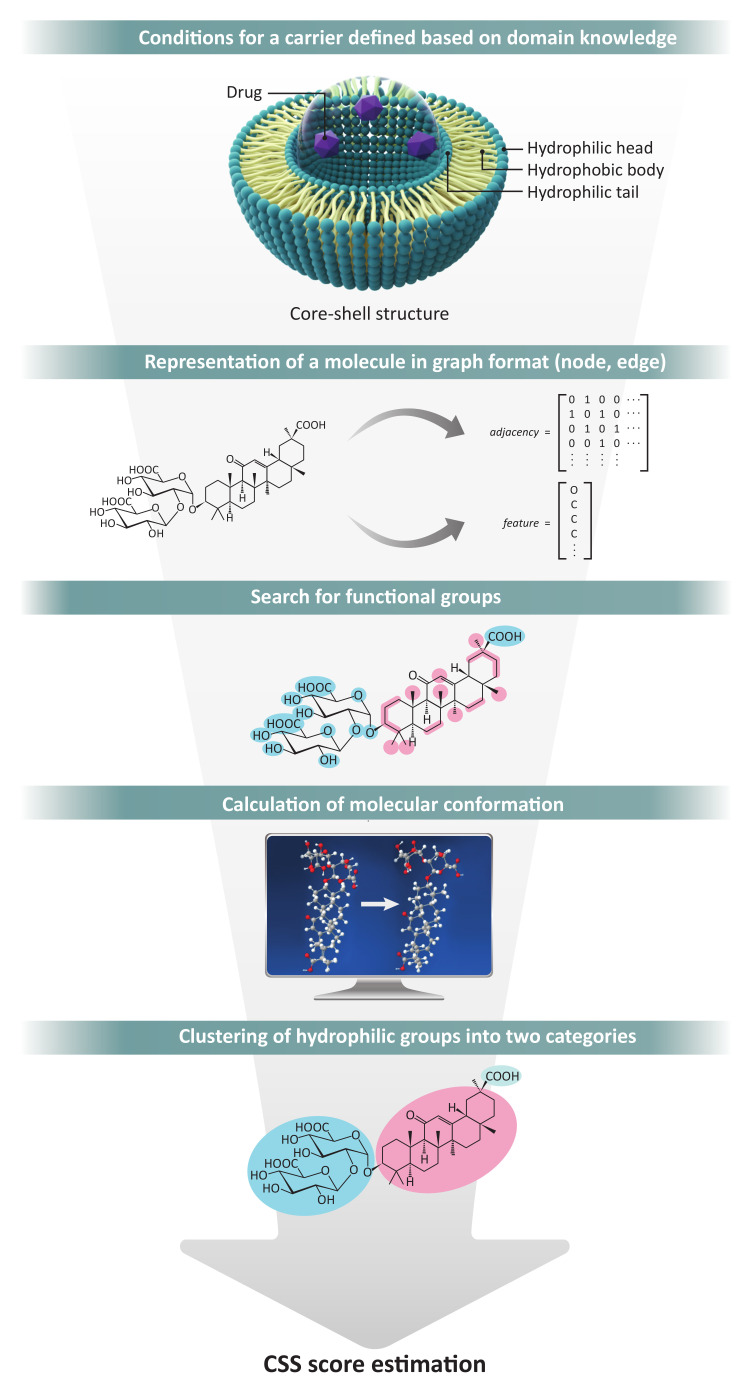
Total process of the CSS method.

The CSS method relies on a canonical simplified-molecular-input line-entry system (SMILES) [17] string to acquire the molobject format from which the molecular conformation is calculated using RDKit (http://www.rdkit.org/, accessed on 8 March 2021) [15]. Molecular conformation refers to the spatial arrangement of the atoms that can be interconverted by gyration of formally single bonds. Subsequently, the molecular graph representation, which is composed of two matrices, atoms and bonds, can be derived from this molecular conformation. Generally, it can be classified as polar or non-polar depending on the property of the chemical group [18]. The CSS method is based on polarity analysis, which requires hydrophilic and hydrophobic groups in the identification of molecules. Each group was detected from the relationship between atoms in molecular graph representation by defining a conditional statement. Hydrophilic groups were clustered into two groups because these groups have to be distinguished by the head and tail. K-means, spectral clustering, and Ward’s hierarchical clustering were used to create a clustering ensemble model for separating objects into two clusters [19,20,21]. Consensus functions for clustering ensemble methods are categorized into two major approaches: object co-occurrence (CO) and median partition. We employed and modified the adaptive clustering ensemble (ACE) method, which is a CO approach [22]. We then built a few algorithms to determine how the molecules meet the pre-defined conditions. Figure 7 provides the total scheme for the CSS method. Single-layer simulation was performed using Schrödinger, which is a physics-based computational platform (https://www.schrodinger.com/, accessed on 8 March 2021) [23]. From the modeling, we identified that a single layer of DB13751is formed between water and nafamostat.

The proposed in silico analysis consists of five processes: Section 3.1 describes the dataset resource and the preprocessing method. Section 3.2 presents a method for finding functional groups in the molecules. Section 3.3 explains the details of the clustering ensemble process. Section 3.4 introduces a method for estimating the suitability scores of molecules. Finally, in Section 3.5, we inspect the validity of our in silico analysis by modeling a single layer of DB13751 between water and nafamostat.

### 3.1. Datasets and Preprocessing

**Figure 8:**
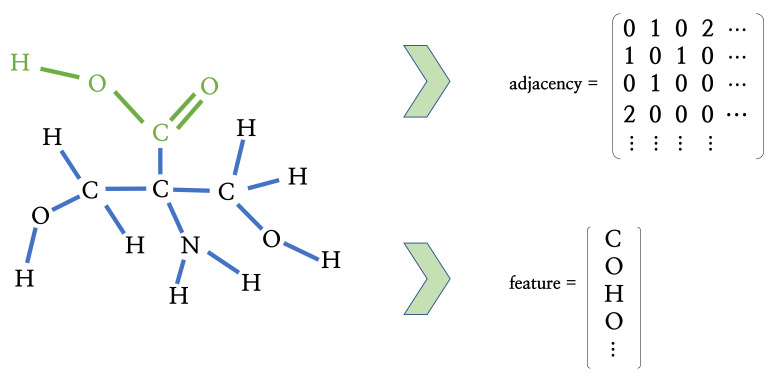
The example of the process representing molecules in graph format. When a molecule has *N* atoms, elements of the adjacency matrix $A\in R^{N\times N}$ denote the bond type between atomic pairs and elements of the feature vector $f\in R^{N\times 1}$, which denotes the atomic numbers. We can see that a carboxylic group (marked in green) is represented in the adjacency matrix and feature vector.

DrugBank is a full, freely web-available resource containing detailed drug, drug action, drug target, and drug–drug interaction information not only about experimental drugs going through the FDA approval process, but also about FDA-approved drugs. In addition, the existing data in DrugBank 5.0 have increased enormously [8]. To limit the molecular search scope, we chose the DrugBank database. Information on 10,630 small molecules, including their canonical SMILES, which is a single-line text representation of a unique molecule, was extracted. RDKit (http://www.rdkit.org/, accessed on 8 March 2021) provides a library converting this canonical SMILES string into mol object format and serves as an indication of the lowest energy molecular conformation and the output coordinates from such a computation [15]. These sets of Euclidean coordinates are used for clustering after a hydrophilic group search. The mol objects can be represented in a molecular graph format, including the adjacency matrix and feature matrix, as shown in Figure 8. The adjacency matrix describes the relationship between atoms called bonds, and the feature matrix consists of atomic numbers.

### 3.2. Identifying Functional Groups in a Molecule

In this section, we discuss the identification of hydrophilic and hydrophobic groups in a molecule. In Davies (1957) [24], the hydrophilic-lipophilic group number was proposed and used to obtain the hydrophilic-lipophilic balance (HLB) value. We employed these groups in [24] as hydrophilic and hydrophobic groups, as shown in Table 2. For example, a carboxylic group (–COOH), marked in green in Figure 8, is classified as a hydrophilic group.

   In the given adjacency matrices and feature vectors of the molecules, we can separate hydrophilic and hydrophobic groups. First, the conditions for a particular functional group are defined. Then, the conditional statement algorithm can be established and applied based on the rules. For example, the process for a carboxylic group search is as follows:

1. The first generation node is “C”. The statement on the feature vector ***f*** is repeatedly applied to search for the root node (first generation node) “C”. Then, the second condition statement will be applied only if the root node corresponds to “C”.
2. “O” is the second-generation node connected to the first-generation node through a single bond. Using the adjacency matrix and feature vectors, we can find the node. The single-bonded atom of the root node is searched for using the adjacency matrix. If a single-bonded atom is found, the atom is allocated as Candidate 1. If Candidate 1 is “O”, the next step will be performed.
3. “H” is the third-generation node connected to the second-generation node through a single bond. Using the adjacency matrix and feature vectors, we can find the node. The single-bonded atom of the second node is searched for using the adjacency matrix. If a single-bonded atom is found, the atom is allocated as Candidate 2. If Candidate 2 is “H”, the next step will be performed.
4. “O” is the second-generation node connected to the first-generation node through a single bond. Using the adjacency matrix and feature vectors, we can find the node. The double-bonded atom of the root node is searched for using the adjacency matrix. If a double-bonded atom is found, the atom is allocated as Candidate 3. If Candidate 3 is “O”, we append the indices of the root nodes, Candidate 1, Candidate 2, and Candidate 3, to the carboxylic group.

**Figure 9:**
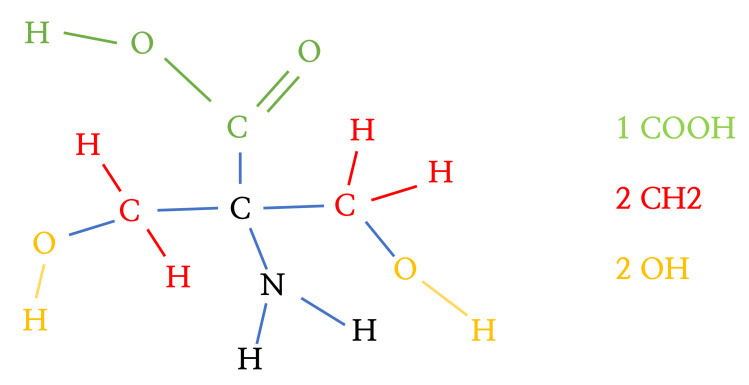
Subtracted groups of molecule.

When all the conditions above are satisfied, the index of the carboxylic group is obtained (Algorithm 1). The condition statement is repeated for each atom. Then, all carboxylic groups within the molecule can be found. In this way, all the hydrophilic and hydrophobic groups that exist within the molecule can be found, as in Figure 9.
**Algorithm 1:** Carboxylic group (–COOH) search.1:**procedure DeleteItem**(adjacency matrix ***A***, feature vector ***f***)2:    **for** *i* in 1 → len(***f***) **do**3:        **if** f[i]= ’C’ **then**4:           candidate1←which(A[i,]==1)5:           **if** f[candidate1]== ’O’) **then**6:               candidate2←which(A[candidate1,]==1)7:               **if** f[candidate2]== ’H’ **then**8:                   candidate3←which(A[i,]==2)9:                   **if** f[candidate3]== ’H’ **then**10:                       cooh.append(list(i, candidate1, candidate2, candidate3))11:    **Return** cooh

### 3.3. Clustering Algorithm

K-means, spectral clustering, and Ward’s hierarchical clustering method were set as clustering members and aggregated using the modified adaptive clustering ensemble (mACE) method. The clustering algorithm consists of five stages: clustering with members, transformation, elimination processes, generating consensus clusters, and enforcing hard clustering, of which all stages except for the first stage are of the mACE algorithm.

#### 3.3.1. The Modified Adaptive Clustering Ensemble

Theoretically, there is no single clustering method that has the best performance due to various disadvantages and a lack of clear guidelines to follow. The sheer variety in protein structure has not allowed for any one clustering method to be sufficient for a given problem. Therefore, many studies have been conducted on clustering ensembles, also referred to as clustering aggregation, which means the process of integrating multiple clustering models (members) into a single amalgamated partition [22].

We adopted and modified the ACE algorithm, one of the clustering ensemble methods, to classify the hydrophilic groups into head and tail [22]. The ACE algorithm has a consensus function based on the object co-occurrence approach. It first calculates the co-occurrence of objects in different members and then decides their cluster labels to generate a consensus result. In other words, it counts the event of an object in one cluster, or that of a pair of objects in the same cluster, and produces the final clustering result by a voting system among the objects. The following sections present how it works in a regular sequence.

Definitions of the mACE algorithm:

1. δ: A binary membership value of an object corresponding to a specific cluster, δ∈{0,1}.
2. θ: The membership matrix, in which the columns are newly formed clusters and the rows are objects.
3. *C*: The set of all the newly formed clusters after the merging process has concluded.
4. $S_{c}$: The cluster similarity $S_{c}$ is an indicator of how much overlap there is between two clusters from different members. Pearson’s correlation values were employed as this indicator and are as follows:
  (1) $$S_{c}\left(c_{j}^{q},c_{j}^{l}\right)=\frac{cov\left(c_{j}^{q},c_{j}^{l}\right)}{\sigma_{c_{j}^{q}}\;\sigma_{c_{j}^{l}}},$$
  where *q* and *l* are the clustering members including clusters *c*.
5. $S_{x}$: The membership similarity $S_{x}$ indicates the similarity between an object $x_{i}$ and a new newly formed cluster $\hat{c}=\left\{c_{i}+c_{j}+\cdots +c_{r}\right\}$ that is formed by summing the initial *r* clusters. It is defined as follows:
  (2) $$S_{x}\left(x_{i},\hat{c_{g}}\right)=\frac{\theta \left(x_{i},\hat{c_{g}}\right)}{max\left\{\theta \left(x_{i},C\right)\right\}},$$
  where *C* denotes the set of newly formed clusters, $C=\left\{\hat{c_{1}},\cdots ,\hat{c_{g}}\right\}$.
6. $\alpha_{1}$: A threshold for merging clusters. Its value is decided based on $S_{c}$.
7. $\alpha_{2}$: A certainty threshold for placing an object into a cluster. Its value is determined based on $S_{x}$.
8. $P_{c}$: Cluster certainty, $P_{c}$, defined as the mean of the membership similarity of objects in a newly formed cluster $\hat{c}$, i.e.,
  (3) $$P_{\hat{c}}=\frac{1}{\left|\hat{c}\right|}\sum_{i=1}^{\left|\hat{c}\right|}S_{x}\left(x_{i},\hat{c}\right).$$
9. $Q_{c}$: Cluster quality, $Q_{c}$, determined as the variance of a newly formed cluster $\hat{c}$, i.e.,
  (4) $$Q_{\hat{c}}=\frac{1}{\left|\hat{c}\right|}\sum_{i=1}^{\left|\hat{c}\right|}{\left(S_{x}\left(x_{i},\hat{c}\right)-P_{\hat{c}}\right)}^{2}.$$
10. Certain object: For an object $x_{i}$, if its maximum membership similarity $S_{x}$ is greater than or equal to a predefined value $a_{2}$, it is seen as a certain object.
  (5) $$\mathrm{if}\;max\left(S_{x}\left(x_{i},C\right)\right)\;\geq \;a_{2},\;\;x_{i}\;\mathrm{is}\;\mathrm{decided}\;\mathrm{to}\;\mathrm{be}\;a\;\mathrm{certain}\;\mathrm{object}.$$
11. Uncertain object: For an object $x_{i}$, if its maximum membership similarity $S_{x}$ is less than a predefined value $a_{2}$, it is seen as a uncertain object.
  (6) $$\mathrm{if}\;max\left(S_{x}\left(x_{i},C\right)\right)\;<\;a_{2},\;\;x_{i}\;\mathrm{is}\;\mathrm{decided}\;\mathrm{to}\;\mathrm{be}\;\mathrm{an}\;\mathrm{uncertain}\;\mathrm{object}.$$

Transformation: After setting *m* members that depict different clustering methods to make initial clusters, this stage transforms them into a new representation. In other words, each cluster *c* is transformed to a binary characteristic vector in which a value of one denotes that the corresponding objects are affiliated with that cluster, and zero denotes the opposite. For a particular *j*th cluster *c* in clustering member *q*, its corresponding vector is expressed as $c_{j}^{q}={\left[\delta \left(x_{1}\right),\cdots ,\delta \left(x_{n}\right)\right]}^{T}$, where $\delta \left(x_{i}\right)$ is the binary membership and takes the following value:

(7) $$\begin{array}{cc} \; & \mathrm{if}\;x_{i}\in c_{j},\;\;\delta \left(x_{i},c_{j}\right)=1,\\ \; & \mathrm{if}\;x_{i}\notin c_{j},\;\;\delta \left(x_{i},c_{j}\right)=0. \end{array}$$

where *i* denotes the index of objects, $j(=1,\ldots ,k_{q})$ denotes the index of clusters in each of *m* members, and q(=1,…,m) represents the index of members in a clustering ensemble. The figure below shows an example of the transformation process.

Generating consensus clusters: The goal here is to find the two most similar clusters and merge them iteratively to generate *k* clusters. For this, the following steps are required:

1. Starting with $k_{m}$ clusters, placed in different members, we measure the cluster similarity $S_{c}$ between the initial clusters. Cluster similarity is defined as Pearson’s correlation, defined in Equation (1).
2. Merge two clusters with the greatest similarity iteratively until no pairs of clusters similar enough remain based on the following criteria:
  (8) $$\mathrm{if}\;S_{c}\left(c_{j}^{q},c_{j}^{l}\right)\;\geq \;a_{1},\;\;c_{j}^{q}\;\mathrm{and}\;c_{j}^{l}\;\mathrm{are}\;\mathrm{merged}\;\left(\mathrm{summed}\right).$$
  (9) $$\mathrm{if}\;S_{c}\left(c_{j}^{q},c_{j}^{l}\right)\;<\;a_{1},\;\;c_{j}^{q}\;\mathrm{and}\;c_{j}^{l}\;\mathrm{are}\;\mathrm{not}\;\mathrm{merged}\;\left(\mathrm{summed}\right).$$

Elimination process: Having generated consensus clusters, if clusters are not merged, the elimination is processed based on cluster certainty. The certainty of each cluster is defined in Equation (3), and their certainty values are sorted in descending order. All the remaining clusters except for the top *k* clusters are eliminated.

1. If the number of newly formed clusters after the consensus function is greater than *k*, a predefined number of clusters in practice, the elimination process is performed.
2. Membership similarity matrix $S_{x}$ between objects and newly formed clusters, defined in Equation (2), is calculated. This is used to enforce assigning each object to the final clusters.
3. Cluster certainty using Equation (3) is computed from membership similarity matrix $S_{x}$. The *k* clusters with the highest cluster certainty values are selected as final consensus clusters.

Enforce hard clustering: In this stage, the aim is to assign each object to just one cluster. Because certain objects have the highest membership similarity value, greater than a2, as defined in Equation (5), it is identified as the corresponding cluster. On the contrary, if an object’s highest membership similarity value is less than a2, it is defined as an uncertain object, as defined in Equation (6). This object can be identified through the minimum effect rule, which is explained in the following process:

1. Identify certain objects in θ as in Equation (5).
2. Estimate the cluster quality of each candidate cluster in θ. The cluster quality, calculated using Equation (4), is defined as the variance of the membership similarity of objects in a cluster.
3. Recalculate the quality of each cluster (candidate) including the membership similarity of the current object (the object to be allocated).
4. Compare the difference between the original cluster quality (Step 2) and the current cluster quality (Step 3).
5. Allocate the current object to the candidate cluster, which has a minimum effect on the original quality.

#### 3.3.2. Implementation of Clustering Algorithms

**Figure 10:**
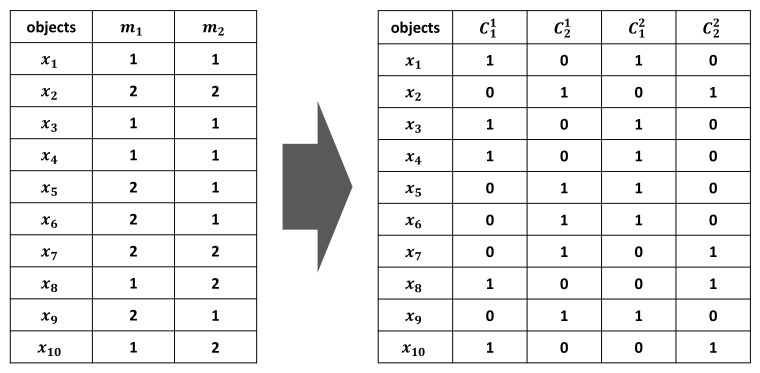
An illustrative example of two clustering members for a dataset with 10 objects.

We employed K-means [19], spectral clustering [20], and Ward’s hierarchical clustering [21] algorithm as members. For the construction phase of clustering members, Scikit-learn (https://scikit-learn.org/, accessed on 8 March 2021) [25], a popular and powerful machine learning library, was used. K-means has been proven to be an effective way to yield good results in clustering problems, but a major drawback of this algorithm is that they would not be used to divide clusters lying on manifolds. Because molecules can be expressed in graph representation, which is viewed as a manifold space consisting of nodes (atoms) and edges (bonds), the spectral clustering and Ward’s clustering algorithm were employed. The number of clusters was set to two, and all parameters were set to default values (Figure 10).

### 3.4. Scoring

**Figure 11:**
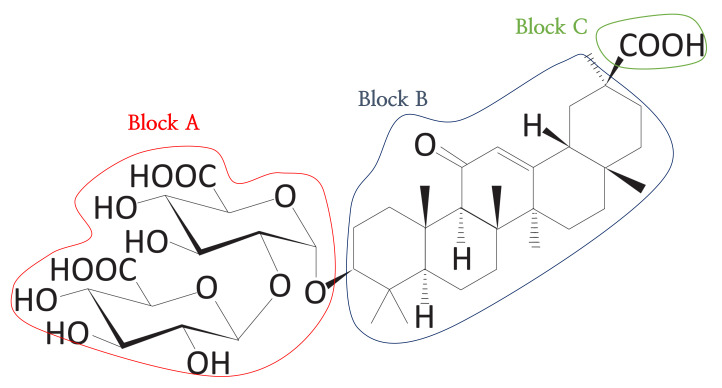
The triblock form of DB13751. The red and green line areas represent the hydrophilic blocks, and the blue line area represents the hydrophobic group derived from the clustering process. We call the red line area Block A, the blue line area Block B, and the green line area Block C. These blocks are used to calculate the sub-scores.

Through a series of processes, a triblock having two hydrophilic blocks and one hydrophobic block was obtained, as shown in Figure 11. There are a total of five sub-scores for obtaining the carrier suitability (CS) score, where four sub-scores are associated with structural properties and one sub-score is related to the value of logP. The following sections describe how to calculate each sub-score.

#### 3.4.1. Score 1: Distance between Blocks A and C

For a molecule to be suitable as a carrier, it should be aligned linearly, and the distance between Blocks A and C should be large. To verify this condition, the distance between the hydrophilic clusters was measured. Score 1 is an indicator of the distance between hydrophilic clusters.

**Figure 12:**
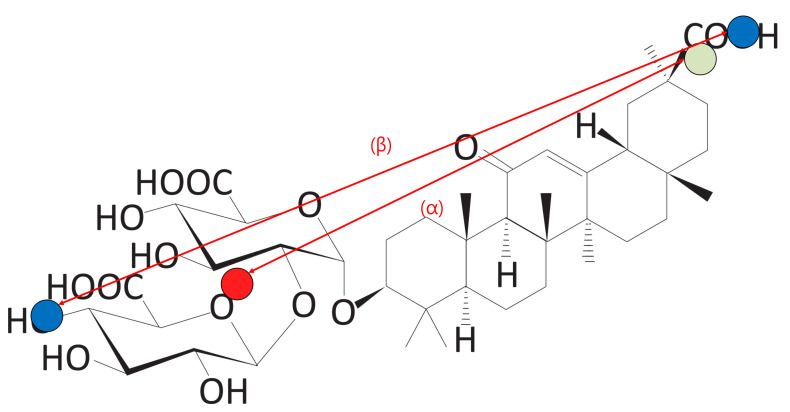
The distance between the hydrophilic clusters (Score 1). Blue dots represent length of the molecule. The red dot represents the center point of Block A. The green dot represents the center point of Block C.

In Figure 12, the red dot is the center point of Block A. The green point is the center point of Block C, and the distance between the points is one. Blue dots represent a head atom and tail atom, respectively. The head atom and the tail atom are the two most distant atoms in the molecule, and the distance between them is referred to the “distance from head to tail”.

If the distance between Blocks A and C (α) is large compared to the total length of the molecule (β), the corresponding molecule is regarded to be highly linear. We define Score 1 as Algorithm 2.
**Algorithm 2: **Score 1: distance between Blocks A and C.1:center point h1 = colMeans(Block A coordinate)2:center point h2 = colMeans(Block C coordinate)3:distance between Blocks A and C = dist(center point h1, center point h2)  4:Score 1 = $\frac{\mathrm{distance}\;\mathrm{between}\;\mathrm{Blocks}\;A\;\mathrm{and}\;C}{\mathrm{distance}\;\mathrm{from}\;\mathrm{head}\;\mathrm{to}\;\mathrm{tail}}$5:**Return** Score 1

#### 3.4.2. Score 2: Within-Block Distance

**Figure 13:**
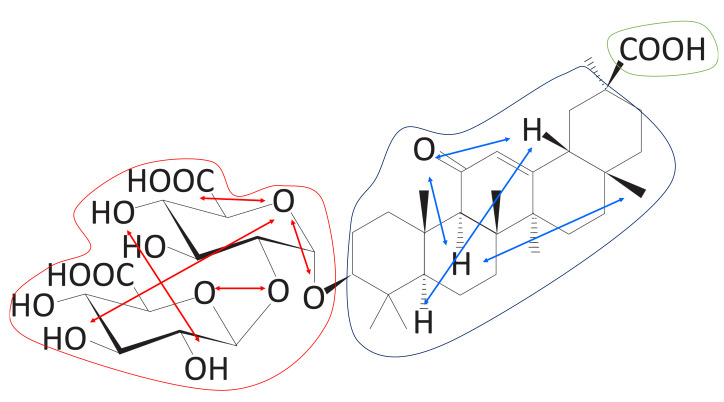
Within-block distance (Score 2). Red arrows represent the distance between groups belonging to Block A. Blue arrows represent the distance between groups belonging to Block B. In this case, Block C has only one group. These distances were used to measure Score 2 (Algorithm 3).

One way to determine if Blocks A, B, and C are clearly separated is to check if each block is internally well packed. In order to determine how well these blocks were packed, the distances between the hydrophilic/hydrophobic groups in each of the blocks were averaged and used as the within-cluster distance. This algorithm is illustrated in Figure 13.
**Algorithm 3: **Score 2: within-block distance.1:h1 = mean(each distance(Block A coordinate))2:h2 = mean(each distance(Block B coordinate))3:h3 = mean(each distance(Block C coordinate))4:final within-group distance = mean(h1, h2, h3)  5:Score 2 = $1-\frac{\mathrm{final}\;\mathrm{within}-\mathrm{group}\;\mathrm{distance}}{\mathrm{distance}\;\mathrm{from}\;\mathrm{head}\;\mathrm{to}\;\mathrm{tail}}$6:**Return** Score 2

#### 3.4.3. Score 3: Size Asymmetry between Block A and Block C

**Figure 14:**
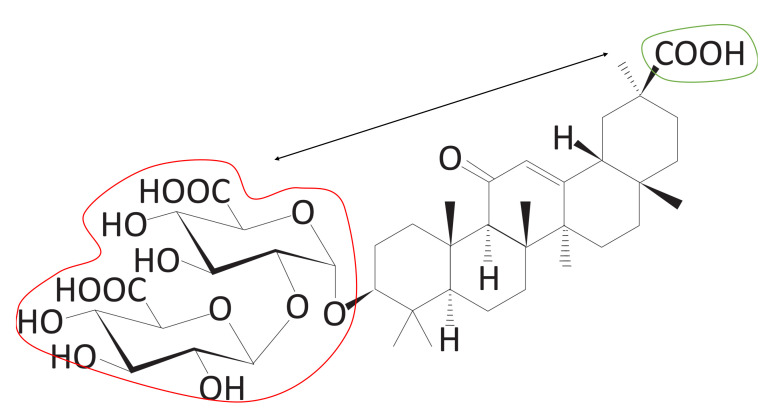
Size asymmetry between Block A and Block C (Score 3). The number of groups belonging to Blocks A and Block C can be compared.

The larger the size difference between Blocks A and C, that is the more the number of groups on one side and the smaller the number of groups on the other, the higher the suitability of the carriers is. This algorithm is illustrated in Figure 14. To calculate this value, the number of groups of Block A and the number of groups in Block C are used. The number of smaller parts of the two is used as a denominator, and the number of larger parts as a numerator in the calculation, which is defined as Score 3. Score 3 is a proportional representation of the size of Blocks A and C (Algorithm 4).
**Algorithm 4: **Score 3: Size asymmetry between Block A and C.1:h1 length = nrow(Triblock A coordinate)2:h2 length = nrow(Triblock C coordinate)  3:Score 3 = $\frac{\max(h1\;\mathrm{length},\;h2\;\mathrm{length})}{min(h1\;\mathrm{length},\;h2\;\mathrm{length})}$4:**Return** Score 3

#### 3.4.4. Score 4: Number of Head and Tail Atoms Included in the Hydrophilic Group

**Figure 15:**
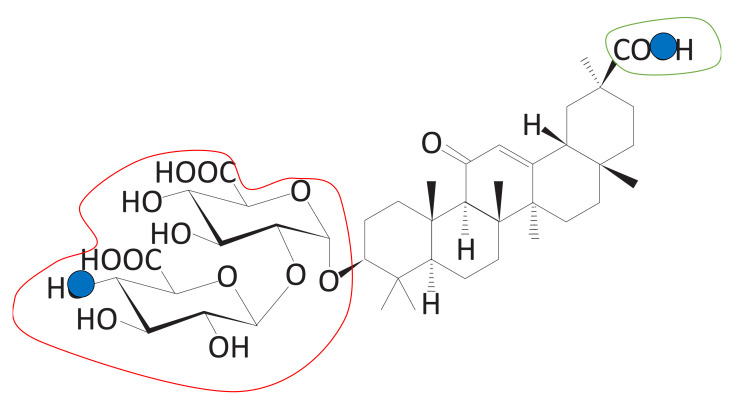
Number of head and tail atoms included in the hydrophilic group (Score 4). The blue dots represent the head and tail atoms, which means two atoms that have the longest distance within a molecule. Head and tail atoms can be checked to determine whether they belong to hydrophilic groups.

If both the head and tail atoms mentioned in Section 3.4.1 are hydrophilic, it can be inferred that the molecules are in the form of blocks and have linearity. Therefore, after finding the head and tail atoms, we identified to which group the molecules belonged. If both molecules belong to hydrophilic groups as in Figure 15, it means that both ends of the molecule are hydrophilic. If only one of the two atoms belongs to a hydrophilic group, only one side of the molecule is a hydrophilic group. If neither atom belongs to a hydrophilic group, it means that the corresponding molecule is less utilizable as a hydrophilic carrier. We define Score 4 as Algorithm 5.
**Algorithm 5: **Score 4: number of head and tail atoms included in the hydrophilic group.1:outermost atom = which.max(each dist(atom coordinate))2:Score 4 = sum(outermost atom ⊂ c(Block A, Block C))3:**Return** Score 4

#### 3.4.5. LogP Score

**Figure 16:**
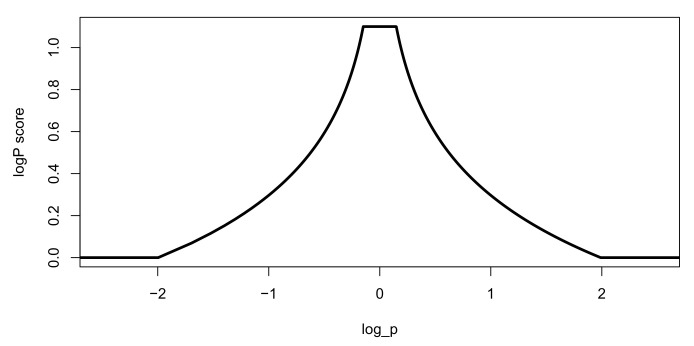
LogP score. The closer the logP value is to zero, the higher the score. The maximum value of the score is 1.1, and the minimum value is zero.

To determine the suitability of a material as a hydrophilic material carrier, it is necessary to consider not only the structural features, but also the chemical properties. Even if hydrophilic groups and hydrophobic groups exist in molecules, if either side is overwhelmingly larger, it is not suitable as a carrier material. Therefore, if the partition coefficient of a molecule is calculated, it can be determined whether the candidate material is suitable. The partition coefficient is an indicator of the hydrophilic/hydrophobic (lipophilic) strength of a molecule; the smaller the value, the more it becomes hydrophilic, and the larger the value, the more hydrophobic. If a particular molecule is excessively hydrophilic or hydrophobic with extreme partition coefficient values, it is deemed unsuitable as a carrier material. Therefore, as the logP value approaches zero, the logP score with a higher score is generated as shown in Figure 16:

(10) $$\mathrm{logP}\;\mathrm{score}=max(min\left(log\left(\frac{2}{|p|+0.01}\right),1.1\right),0).$$

The logP score has a higher value as the molecule is neutral and a lower value as the degree of hydrophilicity or hydrophobicity increases. When logP is zero, it has a maximum value of 1.1, and when logP is lower than −2 or higher than 2, it has a minimum value of zero.

### 3.5. Single Layer Modeling

The Schrödinger software (Version 2018.3) was used to determine whether a single layer of DB13751 can be formed between two materials, especially water and nafamostat. A representative 3D conformation of DB13751 was generated by “LigPrep” of the Schrödinger software. Single layer generation was simulated by “Build Structured Liquid” of the Schrödinger software, with DB13751 between water and nafamostat.

### 3.6. Experimental Methods (Evaluation Methods)

#### 3.6.1. Fabrication of NM-Loaded Micelle NPs

DB13751 (glycyrrhizin, TOKYO CHEMAICAL INDUSTRY, Tokyo, Japan), 20 mg, was dissolved in EtOH 1 ml. Oil (1 mL) was added to the glycyrrhizin solution after checking the layer separation. Simultaneously, nafamostat mesilate (EnzyChem Lifesciences, Seoul, Korea), 4 mg, was dissolved in DW10 mL. The EtOH layer was added to the nafamostat solution, sonicated using a probe-type sonifier, dialyzed using a dialysis membrane bag (MW 6000–8000) and lyophilized. The nafamostat mesilate-loaded micelle NPs are referred to as NM-loaded micelle NPs.

#### 3.6.2. Characterization of NM-Loaded Micelle NPs

The morphology of the glycyrrhizin micelle NPs and NM-loaded micelle NPs was examined using a transmission electron microscope (TEM, Talos L120C, FEI, Prague, Czech) at the National Instrumentation Center for Environmental Management(NICEM). Then, they were individually dispersed in DW (0.1 mg mL${}^{-1}$) to measure their particle size distributions and zeta potential values using a SZ-100V2 instrument (HORIBA Instruments Inc., Kyoto, Japan).

#### 3.6.3. Evaluating the Nafamostat Mesilate Loading Efficient

To evaluate the amount of nafamostat mesilate loaded, the NM-loaded micelle NPs were dissolved in PBS (pH 2), and the loading amount of nafamostat was determined with a plate Reader EPOCH 2 (BioTek Instruments Inc., Winooski, VT, USA) at 260 nm.

#### 3.6.4. Cell Cytotoxicity

A549 cells (human lung adenocarcinoma cell line) were obtained from Korean Cell Line Bank (KCLB, Seoul, Korea). A549 cells were cultured in RPMI 1640 medium (GibcoTM, 22400-089, Waltham, MA, USA) containing 10 % fetal bovine serum (GibcoTM, 16000-044, Waltham, MA, USA) and penicillin–streptomycin solution (GibcoTM, 15140122, Waltham, MA, USA) in an incubator at 37 ${}^{\circ }$C, 5% CO${}_{2}$. A549 cells were inoculated into a 96-well plate at a concentration of 1 × 104 cells/well, and NM-loaded micelle NPs were administered at each concentration (1, 10, 50, 150, and 250 µM). The standard of the concentration was nafamostat. Cells viability analysis after 24 h of incubation was measured with a plate reader at an absorbance of 450 nm using an EZ-Cytox (DoGen, EZ-3000, Suwon, Korea) kit.

## 4. Conclusions

In this study, a novel computational method named CSS for identifying drug carrier candidates using molecular functional groups and their positional information is described. As a condition of screening, whether the hydrophilic cluster has asymmetry, has an amphiphilic structure, or has a linear shape is determined. Six candidates are derived from this method, and one of them is selected through qualitative examination and tested by in vitro experiments. In addition, simulation using the Schrödinger software is performed to examine the formation of a single layer. Consequently, these three tests yield results consistent with those of the computational method we developed. Nevertheless, the research we did has some limitation. First, simulations using the Schrödinger software are used in many computer-aided drug discovery studies and are easy to implement, but are not handled absolutely well. This simulation is just a reference and is not a key validation procedure within this study. Next, the proposed system would be one of many drug screening methods that can include analyses of drug-drug interaction, toxicity prediction, and the drug release profile. Ligand-based drug discovery research such as drug–target binding affinity should be also studied for the prediction of the dual mode of action. Finally, for practical application, the results of in vitro toxicity experiments are presented, but in vitro and in vivo efficacy evaluation experiments should be followed. We propose a method for screening candidates for drug delivery system by analyzing the structure of the molecule. As it is a new approach, there are many limitations, but there would be a great possibility of expanding the research. 

## Figures and Tables

**Table 1:** CS scores of the molecules after qualitative examination. The rows are drugs listed in the DrugBank 5.0 database. The columns show sub-scores that constitute the CS score. Scores 1–4 mean the distance between Block A and C, within-block distance, size asymmetry between Block A and Block C, and number of head and tail atoms included in the hydrophilic group, respectively. The logP score is not the value of logP, and the largest value was regularized when the value of logP was 0. The following sentence indicates the official name of the DB identifier. DB13751:glycyrrhizic acid; DB02675:(4-hydroxymaltosephenyl)glycine; DB05295:eldecalcitol; DB01834:(9R,10R)-9-(S-glutathionyl)-10-hydroxy-9,10-dihydrophenanthrene; DB06543:astaxanthin; DB04150:threonine derivative.

Rank	DB ID	Score 1	Score 2	Score 3	Score 4	logP Score	Total Score
				⋮			
3	DB13751	0.725	0.651	11	2	0	14.403
				⋮			
18	DB02675	0.646	0.703	5.5	2	0	10.118
				⋮			
35	DB05295	0.737	0.746	4	2	0	9.332
				⋮			
74	DB01834	0.582	0.643	5	1	0.632	8.09
				⋮			
82	DB06543	0.997	0.625	1	2	0	8.057
				⋮			
124	DB04150	0.6829	0.65	4	1	0	7.524
				⋮			

**Table 2:** Hydrophilic-hydrophobic group.

Hydrophilic Groups	Hydrophobic Groups
–NR${}_{2}$(tertiary amine)	–CH–
–COO(sorbitan ring)	–CH${}_{2}$–
–COO	–CH${}_{3}$–
–COOH	
–OH	
–O–	
–OH(sorbitan ring)	
–CH${}_{2}$CH${}_{2}$O–	

## Data Availability

The data that support the findings of this study are available from the corresponding author upon reasonable request.

## Abbreviations

The following abbreviations are used in this manuscript:

COVID-19	Coronavirus Disease 2019
DDS	drug delivery system
NM	nafamostat mesilate
NPs	nanoparticles
TEM	transmission electron microscope
DIC	disseminated intravascular coagulation
WHO	World Health Organization
TMPRSS2	transmembrane serine protease 2
DL	deep learning
ML	machine learning
QSPR	quantitative structure–property relationship
SBDD	structure-based drug design
CS	carrier suitability
CSS	carrier suitability scoring
SMILES	simplified-molecular-input line-entry system
CO	co-occurrence
ACE	adaptive clustering ensemble
HLB	hydrophilic-lipophilic balance
mACE	modified adaptive clustering ensemble
NICEM	National Instrumentation Center for Environmental Management
